# NIA Genetics of Alzheimer’s Disease Data Storage Site (NIAGADS)

**DOI:** 10.1002/alz.089040

**Published:** 2025-01-03

**Authors:** Naveensri Saravanan, Amanda B Kuzma, Otto Valladares, Heather Issen Nicaretta, Joseph Manuel, Prabhakaran Gangadharan, Zivadin Katanic, Maureen Kirsch, Youli Ren, Lauren Bass, Jascha Brettschneider, Andrew Wilk, Yi Zhao, Liming Qu, Michelle K Moon, Alexis Lerro Rose, Peter Keskinen, Jeffrey Cifello, Wenhwai Horng, Sam Tate, Flawless Robbins, Heather White, Luke Carter, Shin‐Yi Chou, Adam C. Naj, Emily Greenfest‐Allen, Pavel P. Kuksa, Yuk Yee Leung, Wan‐Ping Lee, Gerard D. Schellenberg, Li‐San Wang

**Affiliations:** ^1^ Penn Neurodegeneration Genomics Center, Philadelphia, PA USA; ^2^ Institute for Biomedical Informatics, Philadelphia, PA USA; ^3^ University of Pennsylvania Perelman School of Medicine, Philadelphia, PA USA; ^4^ Penn Neurodegeneration Genomics Center, Perelman School of Medicine, University of Pennsylvania, Philadelphia, PA USA; ^5^ Institute for Biomedical Informatics, Perelman School of Medicine, Philadelphia, PA USA; ^6^ Penn Neurodegeneration Genomics Center, Dept of Pathology and Laboratory Medicine, University of Pennsylvania, Philadelphia, PA USA; ^7^ Institute for Biomedical Informatics, Perelman School of Medicine, University of Pennsylvania, Philadelphia, PA USA; ^8^ Lehigh University, Bethlehem, PA USA; ^9^ Department of Epidemiology, University of Pennsylvania Perelman School of Medicine, Philadelphia, PA USA; ^10^ Institute on Aging, Philadelphia, PA USA

## Abstract

**Background:**

NIAGADS is a national genomics data repository that facilitates access of genotypic and sequencing data to qualified investigators for the study of the genetics of Alzheimer’s disease (AD) and related neurological diseases. Collaborations with large consortia and centers such as the Alzheimer’s Disease Genetics Consortium (ADGC), Cohorts for Heart and Aging Research in Genomic Epidemiology (CHARGE) Consortium, the Alzheimer’s Disease Sequencing Project (ADSP), and the Genome Center for Alzheimer’s Disease (GCAD) allow NIAGADS to lead the effort in managing large AD datasets that can be easily accessed and fully utilized by the research community.

**Method:**

NIAGADS is supported by the National Institute on Aging (NIA) under a cooperative agreement. All data derived from NIA funded AD genetics studies are expected to be deposited in NIAGADS or another NIA approved site. NIAGADS manages a Data Sharing Service (DSS) that facilitates the deposition and sharing of genomic data and association results with approved users in the neurodegenerative research community. In addition, researchers are able to freely use the NIAGADS Alzheimer’s Genomics Database (www.niagads.org/genomics/) to search annotation resources that link published AD studies to AD‐relevant sequence features and genome‐wide annotations.

**Result:**

As of January 2024, NIAGADS houses 105 datasets comprised of >183,000 samples including array data, sequencing, gene expression, annotations, deep phenotypes, summary statistics, among others. Qualified investigators can retrieve ADSP sequencing data with ease and flexibility through the NIAGADS DSS. To date, the ADSP and other contributing studies have completed whole exome sequencing (WES) of 20,499 samples and whole genome sequencing (WGS) of 36,352 samples. Raw WES and WGS files, quality controlled VCF files, and phenotype data files are available via qualified access. The next round of sequencing currently underway will generate around 30,000 additional genomes to be released in mid‐2024.

**Conclusion:**

NIAGADS is a rich resource for AD researchers, with the goal of facilitating advances in Alzheimer’s genetics research. By housing datasets from many projects and institutions, NIAGADS enables AD researchers to meet their research goals more efficiently. Datasets, guidelines, and features are available on our website at https://www.niagads.org.

